# HHEX Promotes Hepatic-Lineage Specification through the Negative Regulation of Eomesodermin

**DOI:** 10.1371/journal.pone.0090791

**Published:** 2014-03-20

**Authors:** Hitoshi Watanabe, Kazuo Takayama, Mitsuru Inamura, Masashi Tachibana, Natsumi Mimura, Kazufumi Katayama, Katsuhisa Tashiro, Yasuhito Nagamoto, Fuminori Sakurai, Kenji Kawabata, Miho Kusuda Furue, Hiroyuki Mizuguchi

**Affiliations:** 1 Laboratory of Biochemistry and Molecular Biology, Graduate School of Pharmaceutical Sciences, Osaka University, Osaka, Japan; 2 Laboratory of Hepatocyte Differentiation, National Institute of Biomedical Innovation, Osaka, Japan; 3 iPS Cell-Based Research Project on Hepatic Toxicity and Metabolism, Graduate School of Pharmaceutical Sciences, Osaka University, Osaka, Japan; 4 Laboratory of Stem Cell Regulation, National Institute of Biomedical Innovation, Osaka, Japan; 5 Laboratory of Embryonic Stem Cell Cultures, Department of Disease Bioresources Research, National Institute of Biomedical Innovation, Osaka, Japan; 6 Department of Embryonic Stem Cell Research, Field of Stem Cell Research, Institute for Frontier Medical Sciences, Kyoto University, Kyoto, Japan; 7 The Center for Advanced Medical Engineering and Informatics, Osaka University, Osaka, Japan; Wellcome Trust Centre for Stem Cell Research, United Kingdom

## Abstract

Human embryonic stem cells (hESCs) could provide a major window into human developmental biology, because the differentiation methods from hESCs mimic human embryogenesis. We previously reported that the overexpression of hematopoietically expressed homeobox (HHEX) in the hESC-derived definitive endoderm (DE) cells markedly promotes hepatic specification. However, it remains unclear how HHEX functions in this process. To reveal the molecular mechanisms of hepatic specification by HHEX, we tried to identify the genes directly targeted by HHEX. We found that HHEX knockdown considerably enhanced the expression level of eomesodermin (EOMES). In addition, HHEX bound to the HHEX response element located in the first intron of EOMES. Loss-of-function assays of EOMES showed that the gene expression levels of hepatoblast markers were significantly upregulated, suggesting that EOMES has a negative role in hepatic specification from the DE cells. Furthermore, EOMES exerts its effects downstream of HHEX in hepatic specification from the DE cells. In conclusion, the present results suggest that HHEX promotes hepatic specification by repressing EOMES expression.

## Introduction

The molecular mechanisms of liver development have been clarified by using model organisms such as chicks, *Xenopus*, *zebrafish*, and mice [1]–[2]. Although these models have many advantages, the molecular mechanisms of human liver development might be different from those of model organisms. The use of differentiation models from human embryonic stem cells (hESCs) for studying human development might resolve these problems, because these differentiation methods mimic human embryogenesis [3]. Previous reports have demonstrated that the definitive endoderm (DE) cells could be efficiently generated from hESCs in the presence of Activin A [4], and that the hESC-derived DE cells have the potential to differentiate into various DE-derived lineages, such as hepatocytes, pancreatic beta-cells, and small intestinal enterocytes[5]–[7]. In hepatic differentiation, Agarwal et al. reported that the typical gene expression profiles observed in the differentiation model from hESCs are similar to those observed in fetal liver development [8]. In addition, we previously reported that CCAAT/enhancer binding protein-mediated regulation of TGF beta receptor 2 expression determines the hepatoblast fate decision by using a differentiation model from hESCs [9]. The use of differentiation models from hESCs, rather than the usual model organisms, would provide great opportunities to expand our understanding of the molecular mechanisms.

A transcription factor, *hematopoietically expressed homeobox* (HHEX), is initially expressed in DE, and then its expression is restricted to the future hepatoblasts, which could segregate into both hepatocytes and cholangiocytes [10]. In the *HHEX*-null embryo, some hepatic gene expression levels are reduced and further hepatic development is prevented [11]–[12]. These studies indicate that the transcription factor HHEX plays an essential role in hepatic specification from DE. Recently, we reported that overexpression of HHEX by using adenovirus (Ad) vectors in the hESC-derived DE cells markedly promotes the hepatic specification [13]. Moreover, Kubo et al. demonstrated that HHEX promotes this process by synergistically working with bone morphogenetic protein 4 (BMP4), and they expected that HHEX might function with *HNF1 homeobox A* (HNF1α) [14], which is known to be its co-activator [15]. However, the functions of HHEX in this process are not well understood, and the target genes of HHEX have not been investigated in detail. Therefore, we attempted to identify the target genes of HHEX in the hepatic specification by using a differentiation model from hESCs.

In the present study, to elucidate the functions of HHEX in hepatic specification from DE, we attempted to identify the target genes of HHEX by using the hepatic differentiation model from hESCs. To this end, the candidate target gene of HHEX were verified by performing ChIP-qPCR and luciferase reporter assays, and then loss-of-function assays were performed to clarify the functions of the candidate target gene in the hepatic specification. These results confirmed that *eomesodermin* (EOMES), which is known to regulate DE differentiation, is one of the crucial target genes of HHEX in human hepatic specification from the DE. Our report thus shows for the first time that HHEX promotes hepatic specification through the repression of EOMES expression.

## Materials and Methods

### hESCs Culture

A hESC line, H9 (WA09, WISC Bank, WiCell Research Institute), was maintained on a feeder layer of mitomycin C-treated mouse embryonic fibroblasts (MEF) (Millipore) with ReproStem medium (ReproCELL) supplemented with 5 ng/ml fibroblast growth factor 2 (FGF2) (KATAYAMA CHEMICAL INDUSTRIES). hESCs were dissociated with 0.1 mg/ml dispase (Roche) into small clumps and then were subcultured every 4 or 5 days. H9 was used following the Guidelines for Utilization of Human Embryonic Stem Cells of the Ministry of Education, Culture, Sports, Science and Technology of Japan after approval by the institutional ethical review board at National Institute of Biomedical Innovation.

### *In vitro* Differentiation

The differentiation protocol for the induction of DE cells and hepatoblasts was based on our previous report with some modifications [13–16–21]. Briefly, hESCs were dissociated by using dispase and suspended in MEF-conditioned ReproStem medium supplemented with 10 ng/ml FGF2, and then plated onto a growth factor reduced Matrigel (BD Biosciences)-coated dish. When hESCs reached approximately 80% confluence, the MEF-conditioned ReproStem medium was replaced with the differentiation RPMI-1640 medium (Sigma) containing 100 ng/ml Activin A (R&D systems) (the differentiation RPMI-1640 medium is consisted with RPMI-1640 medium (Sigma) supplemented with B27 supplement (Invitrogen) and 4 mM L-glutamine), and then cultured for 4 days. For induction of the hepatoblasts, the DE cells were cultured for 5 days in the differentiation RPMI-1640 medium supplemented with 20 ng/ml BMP4 (R&D Systems) and 20 ng/ml FGF4 (R&D Systems).

### RNA Isolation and Reverse Transcription-PCR

Total RNA was isolated from hESCs and their derivatives using ISOGENE (Nippon Gene). cDNA was synthesized using 500 ng of total RNA with a SuperScript VILO cDNA Synthesis Kit (Invitrogen). Real-time RT-PCR was performed with SYBR Green PCR Master Mix (Applied Biosystems) using an Applied Biosystems StemOnePlus real-time PCR systems. Relative quantification was performed against a standard curve and the values were normalized against the input determined for the housekeeping gene, glyceraldehyde 3-phosphate dehydrogenase (GAPDH). The primer sequences used in this study are described in **Table S1 in File S2**.

### Flow Cytometry

Single-cell suspensions of the hESC derivatives were fixed with 2% paraformaldehyde (PFA) at 4°C for 20 minutes and then incubated with the primary antibody, followed by the secondary antibody. Flow cytometry analysis was performed using a FACS LSR Fortessa flow cytometer (BD Biosciences). All the antibodies are listed in **Table S2 in File S2**.

### ChIP-qPCR

ChIP assays were performed by using a Chromatin Immunoprecipitation Assay Kit (Millipore) according to the manufacturer’s instructions. The hESC-derived cells (approximately 1.0 × 10^6^ cells) were cross-linked with 1% formaldehyde at room temperature for 10 minutes. The cells were washed once with PBS containing protease inhibitors (1 mM phenylmethylsulfonyl fluoride, 1 mg/ml aprotinin and 1 mg/ml pepstatin A) and then harvested using a cell scraper. The cross-linked cells were centrifuged and resuspended with sodium dodecyl sulfate (SDS) lysis buffer with the protease inhibitors described above, and then incubated on ice for 10 minutes. The cells were sonicated to solubilize and shear cross-linked DNA. The resulting whole cells were centrifuged, and the supernatants were diluted in ChIP Dilution Buffer containing the protease inhibitors described above, then added to Protein A magnetic beads and rotated at 4°C for 30 minutes. Next, the supernatants of these cells were immunoprecipitated with anti-human HHEX antibody (Santa Cruz Biotechnology, sc-15129) or anti-goat IgG antibody at 4°C overnight with rotation. On the following day, the resulting supernatants were added to Protein A magnetic beads and rotated at 4°C for 60 minutes, then washed five times with Low Salt Immune Complex Wash Buffer (one time), High Salt Immune Complex Wash Buffer (one time), LiCl Immune Complex Wash Buffer (one time), and TE Buffer (two times) for 5 minutes per wash with rotation. Bound complexes were added to elution buffer (1% SDS, 0.1 M NaHCO_3_) at room temperature for 15 minutes with rotation, and then the supernatants were added to 5 M NaCl and were eluted at 65°C for 4 hours. Immunoprecipitated DNA was purified by treatment with 0.5 M EDTA, 1 M Tris-HCl, and 10 mg/ml proteinase K at 45°C for 60 minutes and recovered by phenol/chloroform alcohol extraction and ethanol precipitation. Purified DNA was used as a template for qPCR according to the protocol described in the *RNA isolation and reverse transcription-PCR* section above. All the antibodies are listed in **Table S2 in File S2**. The primer sequences used in this study are described in **Table S1 in File S2**.

### Plasmid Constructions

The promoter region of EOMES was cloned. To generate the 5′ untranslated region (UTR) of the EOMES-firefly luciferase reporter construct (pGL3-EOM-5UTR1000), a 1,000 bp 5′ UTR of the human EOMES was amplified by using the following primers: 5′-AGCGGTACCTTCCTCTCTACAAACCTTTCCCACTGGG-3′ and 5′-TAACCATGGGCTTTGCAAAGCGCAGACGGCAGCTGGCTGC-3′ (−1,000/−1 5′ UTR of EOMES; KpnI and NcoI restriction sites incorporated into sense and antisense primers, respectively, are underlined) and to generate the long 5′ UTR of the EOMES-firefly luciferase reporter construct (pGL3-EOM-5UTR4000), a 4,000 bp 5′ UTR of the human EOMES was amplified by using the following primers: 5′-CAGGGTACCGATAACACGTTTTTAGTGGGGGTG-3′ and 5′-TAACCATGGGCTTTGCAAAGCGCAGACGGCAGCTGGCTGC-3′ (−4,000/−1−5′ UTR of EOMES; KpnI and NcoI restriction sites incorporated into sense and antisense primers, respectively, are underlined). Each 5′ UTR of the human EOMES was cloned into the promoter region of the pGL3-Basic vector (Promega) using KpnI and NcoI restriction sites. In addition, the 400 bp region around the HHEX response element (HRE) was amplified by using the following primers: 5′-CCTGCTAGCGTTCTCTGGTACTTTTCAAAATGGTGC-3′ and 5′-GAAAACTAGTATGCGCCTGTGCAAGGGAATAGAATCAG-3′. The 400 bp region around the HRE was cloned into the enhancer region of each of pGL3-EOM-5UTR1000 and pGL3-EOM-5UTR4000 using XbaI restriction site to generate pGL3-EOM-5UTR1000 containing the region around the HRE (p5’ EOM-Luc) and pGL3-EOM-5UTR4000 containing the region around the HRE (pLong-5′ EOM-Luc).

To generate pGL3-EOM-5UTR1000 containing the region which has a mutated HRE reporter construct (p5’ EOM-mut-Luc), the following base substitutions were introduced into the 400 bp region around the HRE: 5′-TCCCAATTAAAATC-3′ to 5′-TCC***AGC***T***G***A***C***AATC-3′. PCR products were cloned into the enhancer region of pGL3-EOM-5UTR1000 using XbaI restriction site.

### Luciferase Reporter Assays

HeLa cells were transfected with each of the firefly luciferase reporter plasmids described above (p5’ EOM-Luc or p5’ EOM-mut-Luc) or control plasmids, pGL3-Basic vector plasmids (pControl-Luc), by using Lipofectamine 2000 (Invitrogen)-mediated gene transfection according to the manufacturer’s instructions. HeLa cells were seeded at a density of 2.0 × 10^5^ cells/well in 24-well tissue culture plates, and cultured for 24 hours before transfection. HeLa cells were transfected with 333 ng/well of each firefly luciferase reporter plasmids (pControl-Luc, p5’ EOM-Luc, or p5’ EOM-mut-Luc), 333 ng/well of HHEX expression plasmids (pHMEF5-HHEX [13]) or blank expression plasmids (pHMEF5), and 333 ng/well of internal control plasmids (pCMV-Renilla luciferase), and cultured for 72 hours. The luciferase activities in the cells were measured by using Dual Luciferase Assay System (Promega) according to the manufacturer’s instructions. Firefly luciferase activities in the cells were normalized by the measurement of renilla luciferase activities. The luciferase activity in the cells cotrasfected with pControl-Luc and pHMEF5 was assigned a value of 1.0.

### siRNA Transfection

Knockdown of HHEX or EOMES was performed using a specific small interfering RNA (siRNA) fourplex set targeted to HHEX or EOMES, respectively (Darmacon SMARTpool) (Thermo Fisher Scientific). Si-Control (Darmacon siGENOME Non-Targeting siRNA Pool) (Thermo Fisher Scientific) was used as a control. Lipofectamine RNAiMAX (Invitrogen)-mediated gene transfection was used for the reverse transfection according to the manufacturer’s instructions. The hESC-derived DE cells on day 4 were transfected with 50 nM of siRNA for 6 hours by reverse transfection.

### Immunohistochemistry

The hESC-derived cells were fixed with methanol or 4% PFA. After blocking with PBS containing 1% BSA (Sigma), 0.2% Triton X-100 (Sigma), and 10% FBS, the cells were incubated with primary antibody at 4°C overnight, followed by incubation with a secondary antibody that was labeled with Alexa Fluor 488 (Invitrogen) at room temperature for 1 hour. All the antibodies are listed in **Table S2 in File S2**.

### Western Blotting Analysis

The hESC-derived cells were homogenized with lysis buffer (20 mM HEPES, 2 mM EDTA, 10% glycerol, 0.1% SDS, 1% sodium deoxycholate, and 1% Triton X-100) containing a protease inhibitor mixture (Sigma). After being frozen and thawed, the homogenates were centrifuged at 15,000 *g* at 4°C for 10 minutes, and the supernatants were collected. The lysates were subjected to SDS-PAGE on 7.5% polyacrylamide gel and were then transferred onto polyvinylidene fluoride membranes (Millipore). After the reaction was blocked with 1% skim milk in TBS containing 0.1% Tween 20 at room temperature for 1 hour, the membranes were incubated with anti-human HHEX, EOMES, or β-actin antibodies at 4°C overnight, followed by reaction with horseradish peroxidaseconjugated anti-rabbit IgG or anti-mouse IgG antibodies at room temperature for 1 hour. The band was visualized by ECL Plus Western blotting detection reagents (GE Healthcare) and the signals were read using an LAS-4000 imaging system (Fuji Film). All the antibodies are listed in **Table S2 in File S2**.

## Results

### Obstruction of Hepatoblast Differentiation by HHEX Knockdown Results in Upregulation of the Expression Levels of DE Markers

**Figure 1:**
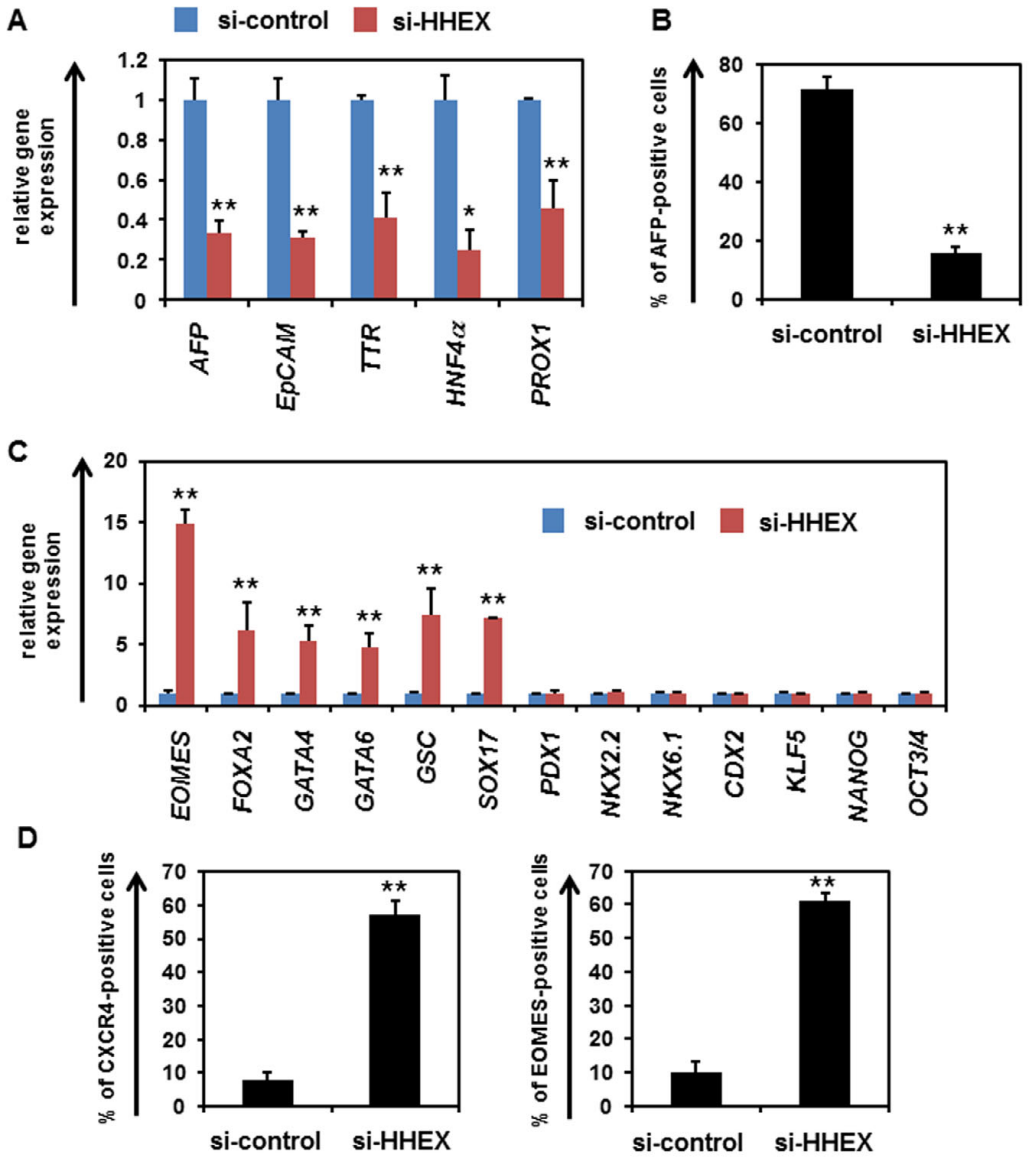
The expression levels of DE markers in the si-HHEX-transfected cells were upregulated in hepatoblast differentiation from DE cells. (**A**) hESCs (H9) were differentiated into DE cells according to the protocol described in the *Materials and Methods* section. The DE cells were transfected with 50 nM si-control or si-HHEX on day 4, and cultured in the medium containing 20 ng/ml BMP4 and 20 ng/ml FGF4 until day 9. On day 9, the gene expression levels of hepatoblast markers (*AFP*, *EpCAM*, *TTR*, *HNF4α*, and *PROX1*) in si-control- or si-HHEX-transfected cells were examined by real-time RT-PCR. The gene expression levels in the si-control-transfected cells were taken as 1.0. (**B**) On day 9, the percentage of AFP-positive cells was measured by using FACS analysis to examine the hepatoblast differentiation efficiency. (**C**) The gene expression levels of DE (*EOMES*, *FOXA2*, *GATA4*, *GATA6*, *GSC*, and *SOX17*), pancreatic (*PDX1*, *NKX2.2*, and *NKX6.1*), intestinal (*CDX2* and *KLF5*), and pluripotent markers (*NANOG* and *OCT3/4*) in the si-control- or si-HHEX-transfected cells were examined by real-time RT-PCR. The gene expression levels in the si-control-transfected cells were taken as 1.0. (**D**) On day 9, the percentage of cells positive for the DE markers (CXCR4 and EOMES) was examined by using FACS analysis. All data are represented as means ± SD (*n = *3). *p<0.05, **p<0.01.

It is known that HHEX plays an important role in hepatoblast differentiation [11–12–14]. We have previously reported that HHEX overexpression promoted hepatoblast differentiation from the hESC-derived DE cells [13]. To confirm the importance of HHEX in hepatoblast differentiation, a loss of function assay of HHEX was performed by using siRNA-mediated HHEX knockdown. We confirmed the knockdown of HHEX expression in the hESC-derived DE cells that has been transfected with si-HHEX (**Fig. S1 in File S1)**. The gene expression levels of hepatoblast markers in the si-HHEX-transfected cells were significantly downregulated as compared with those in the si-control-transfected cells (**Fig. 1A**). In addition, the percentage of alpha-fetoprotein (AFP; a hepatoblast marker)-positive cells was decreased by HHEX knockdown on day 9 (**Fig. 1B**). These results suggest that hepatoblast differentiation is prevented by HHEX knockdown, demonstrating that HHEX plays an important role in hepatoblast differentiation from DE cells. To characterize the si-HHEX-transfected cells on day 9, the gene expression levels of DE, pancreatic, intestinal, and pluripotent markers were examined (**Fig. 1C**). Interestingly, the gene expression levels of DE markers were significantly upregulated by HHEX knockdown, although those of pancreatic, intestinal, and pluripotent markers were not changed by HHEX knockdown. Furthermore, the percentage of DE marker (CXCR4 and EOMES)-positive cells was increased by HHEX knockdown (**Fig. 1D**). In addition, the percentage of AFP-positive cells or *EOMES* expression level was decreased or increased, respectively, by HHEX knockdown not only in the DE cells (day 4) but also in the cells starting to commit to hepatoblast (day 5–7) (**Fig. S2 in File S1**). This suggested that HHEX knockdown inhibits hepatoblast differentiation but does not simply change the number of the DE cells. These results suggest that the inhibition of HHEX expression during hepatoblast differentiation results in an increase of DE cells, but not pancreatic, intestinal, or undifferentiated cells.

### HHEX Directly Represses EOMES Expression

**Figure 2:**
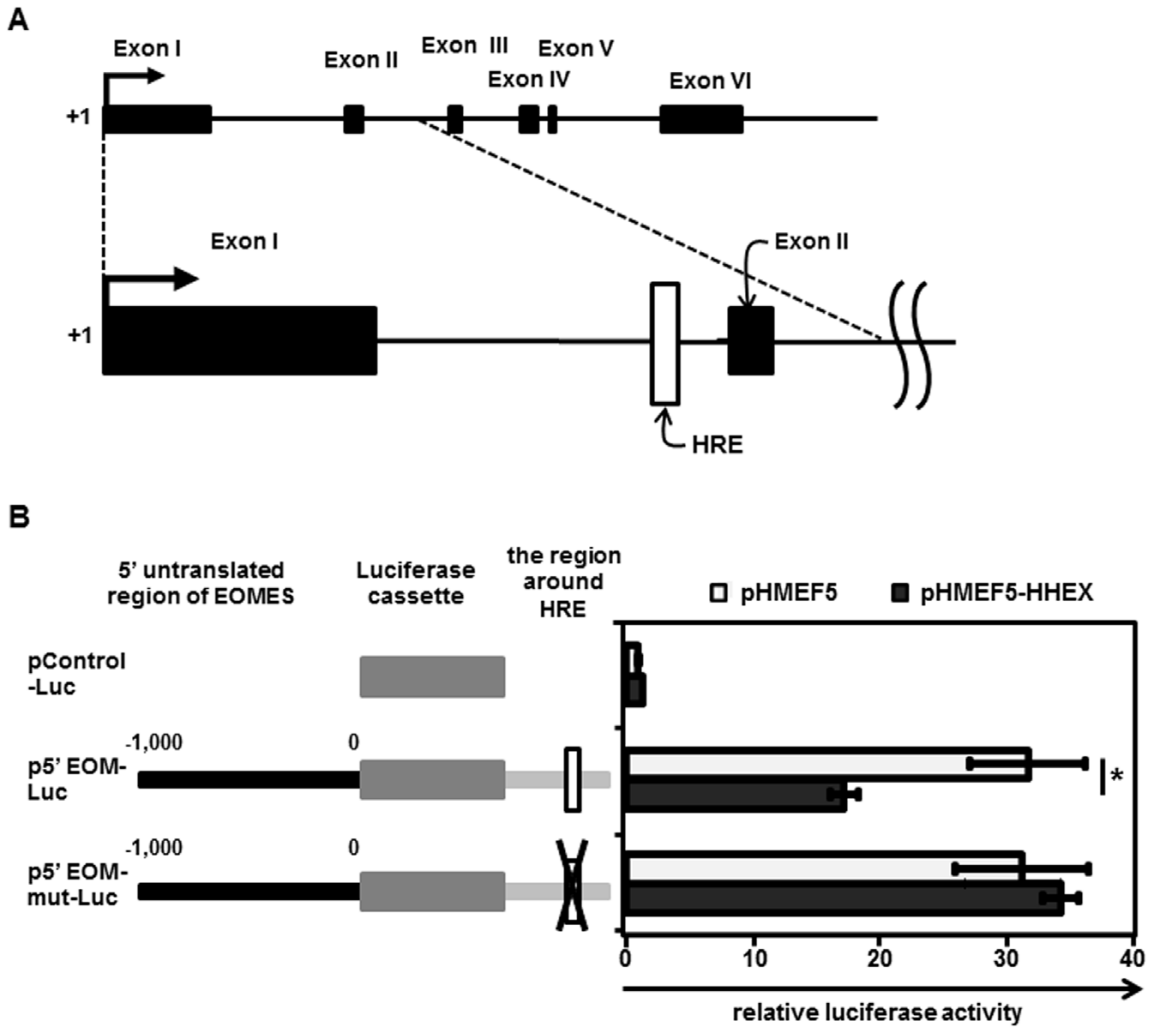
HHEX suppresses EOMES expression by binding to the HRE located in the first intron of EOMES. (**A**) An overview of the EOMES mRNA precursor and the location of the putative HRE are presented. The HRE is located in the first intron of EOMES. (**B**) Luciferase reporter assays were performed to examine the regulation of EOMES expression by HHEX. HeLa cells were cotransfected with both firefly luciferase reporter plasmids (pControl-Luc, p5’ EOM-Luc, or p5’ EOM-mut-Luc) and effecter plasmids (control plasmids (pHMEF5) or HHEX expression plasmids (pHMEF5-HHEX)). The details of the luciferase reporter assays are described in the *Materials and Methods* section. The luciferase activities in the pControl-Luc- and pHMEF5-cotransfected cells were taken as 1.0. All data are represented as means ± SD (*n = *3). *, *p*<0.05.

Because the gene expression level of *EOMES* was most increased by HHEX knockdown in hepatoblast differentiation, we expected that EOMES might be directly regulated by HHEX. The putative HHEX-binding site (HHEX response element (HRE)) [22] was found in the first intron of EOMES as shown in **Figure 2A**. To investigate whether HHEX could directly repress EOMES transcription, luciferase reporter assays were performed. The reporter plasmids that contain a 5′ untranslated region (UTR) of EOMES (**Fig. S3 in File S1**) and the first intron of EOMES were generated because the putative HHEX-binding site was observed in the first intron of EOMES. The luciferase reporter assays showed that p5’ EOM-Luc, which contains the wild-type HRE, mediates significant repression of luciferase activity by HHEX overexpression, whereas p5’ EOM-mut-Luc, which contains a mutant HRE, mediates similar luciferase activity even in the presence of HHEX (**Fig. 2B**). These results indicated that HHEX represses EOMES expression through the HRE located in the first intron of EOMES.

### Endogenous Temporal Gene Expression Analysis of HHEX and EOMES in Hepatic Specification

**Figure 3:**
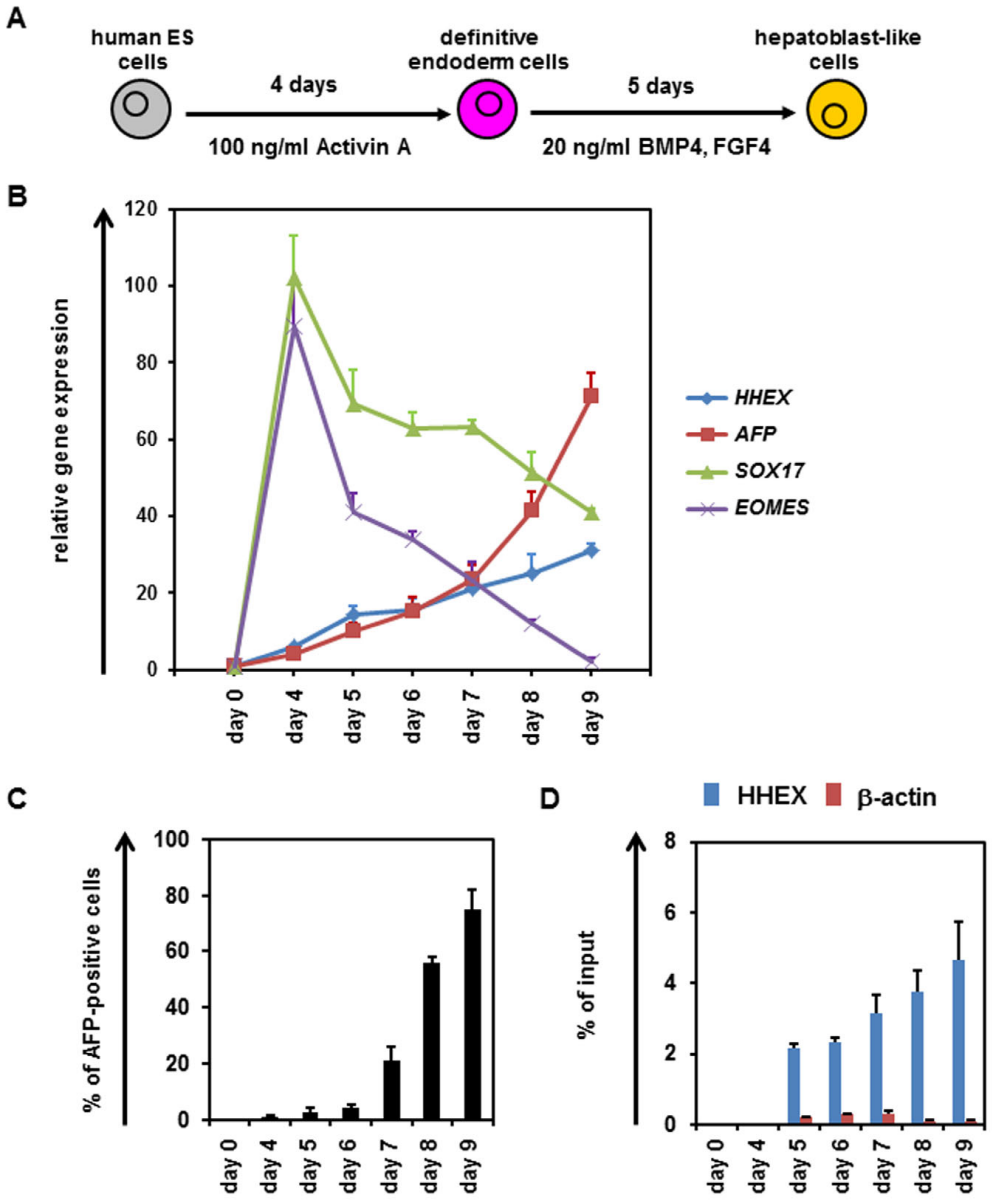
Temporal analysis of endogenous gene expression levels of EOMES and HHEX in hepatoblast differentiation from hESCs. (**A**) The schematic protocol for hepatoblast differentiation from hESCs (H9) is shown. (**B**) The temporal gene expression levels of *HHEX*, *AFP*, *SOX17* and *EOMES* were examined by real-time RT-PCR in hepatoblast differentiation. The gene expression levels in undifferentiated hESCs were taken as 1.0. (**C**) To examine the hepatoblast differentiation efficiency, the percentage of AFP-positive cells was measured by FACS analysis. (**D**) The HHEX protein-binding frequencies of the regions around the HRE of the EOMES gene and a negative control gene (β-ACTIN) were measured by ChIP-qPCR analysis. The results are presented as the percent input of anti-HHEX samples compared with those of anti-IgG samples. All data are represented as means ± SD (*n = *3).

To examine the relationship between HHEX and EOMES in hepatic specification, the temporal gene expression patterns of *HHEX* and *EOMES* were examined in hepatoblast differentiation from hESCs (**Fig. 3A**). In DE differentiation (from day 0 to 4), the gene expression levels of *EOMES* and *SOX17* were increased, although those of *HHEX* and *AFP* did not change (**Fig. 3B**). In the hepatic specification process (from day 5 to 9), the gene expression levels of *HHEX* and *AFP* began to be upregulated on day 5, and continued to increase until day 9. On the other hand, the gene expression levels of *EOMES* and *SOX17* started to decrease on day 5, and continued to decrease until day 9. We confirmed that the percentage of CXCR4-positive cells was 95.2±2.2% on day4. In addition, we confirmed that few AFP-positive cells were observed on day 5, and that the percentage of AFP-positive cells continuously increased until day 9 (**Fig. 3C**). To examine whether HHEX binds to the HRE located in the first intron of EOMES, ChIP-qPCR analysis of hepatoblast differentiation from hESCs was performed (**Fig. 3D**). HHEX bound to the HRE located in the first intron of EOMES on day 5, when the hepatic specification began. The amount of HHEX binding to that site continued to increase until day 9. These results suggest that HHEX binds to HRE located in the first intron of EOMES in hepatic specification from the DE cells.

### EOMES Knockdown Promotes Hepatic Specification in the Presence of BMP4

**Figure 4:**
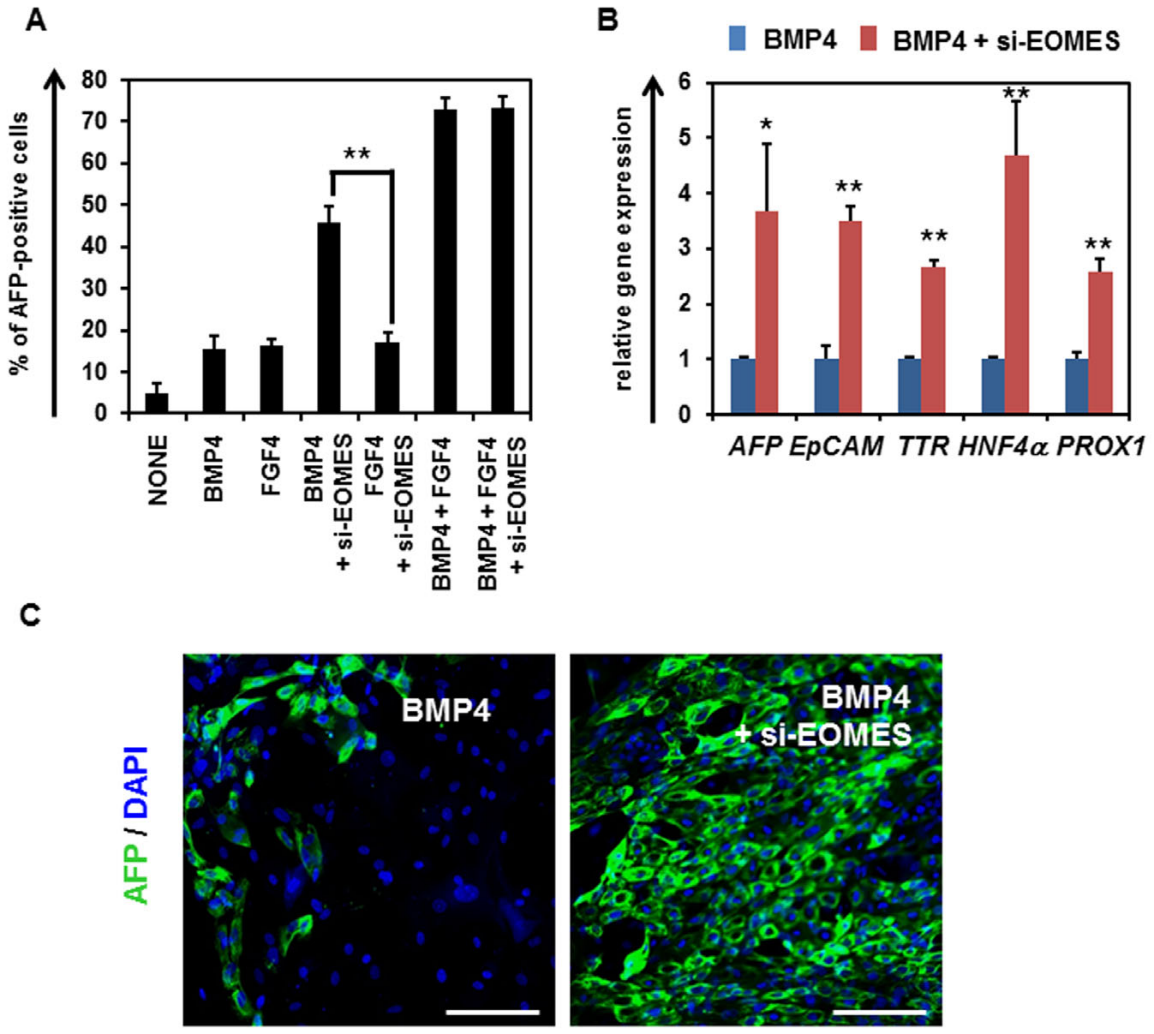
Hepatoblast differentiation was promoted by knockdown of EOMES in the presence of BMP4. (**A**) The hESCs (H9) were differentiated into the DE cells according to the protocol described in the *Materials and Methods* section. The hESC-derived DE cells were transfected with 50 nM si-control or si-EOMES on day 4, and then cultured with the medium containing BMP4 or FGF4. The percentage of AFP-positive cells was examined by FACS analysis on day 9. (**B**) The gene expression levels of hepatoblast markers (*AFP*, *EpCAM*, *TTR*, *HNF4α*, and *PROX1*) were measured by real-time RT-PCR on day 9. The gene expression levels in si-control-transfected cells were taken as 1.0. (**C**) The si-control- or si-EOMES-transfected cells were subjected to immunostaining with anti-AFP (green) antibodies. Nuclei were counterstained with DAPI (blue). The bar represents 50 μm. All data are represented as means ± SD (*n = *3). *p<0.05, **p<0.01.

To examine the function of EOMES in hepatoblast differentiation, EOMES was knocked down in the DE cells in the presence of BMP4 or FGF4. We confirmed the knockdown of EOMES expression in the hESC-derived DE cells that has been transfected with si-EOMES (**Fig. S4 in File S1)**. Although the percentage of AFP-positive cells was increased by EOMES knockdown in the presence of BMP4, it was not changed by EOMES knockdown in the presence of FGF4 (**Fig. 4A**). In addition, EOMES knockdown did not affect the percentage of AFP-positive cells in the presence of both FGF4 and BMP4. This might have been because the endogenous *EOMES* expression level was already sufficiently suppressed under the existence of FGF4 (**Fig. S5 in File S1**). To further investigate the function of EOMES in hepatoblast differentiation, gene expression and immunohistochemical analyses of hepatoblast markers were performed in si-EOMES-transfected cells. The gene expression levels of hepatoblast markers in si-EOMES-transfected cells were upregulated as compared with those in si-control-transfected cells (**Fig. 4B**). Consistently, the immunohistochemical analysis of AFP showed that EOMES knockdown upregulated the expression levels of AFP (**Fig. 4C**). In addition, EOMES knockdown increased the percentage of AFP-positive cells not only in the DE cells (day 4) but also in the cells starting to commit to hepatoblast (day 5–7) (**Fig. S6 in File S1**). This suggested that EOMES knockdown promotes hepatoblast differentiation but does not simply change the number of the DE cells. These results suggest that hepatic specification from the DE cells is promoted by EOMES knockdown depending on the existence of BMP4.

### EOMES Functions Downstream of HHEX in the Hepatic Specification from the DE Cells

**Figure 5:**
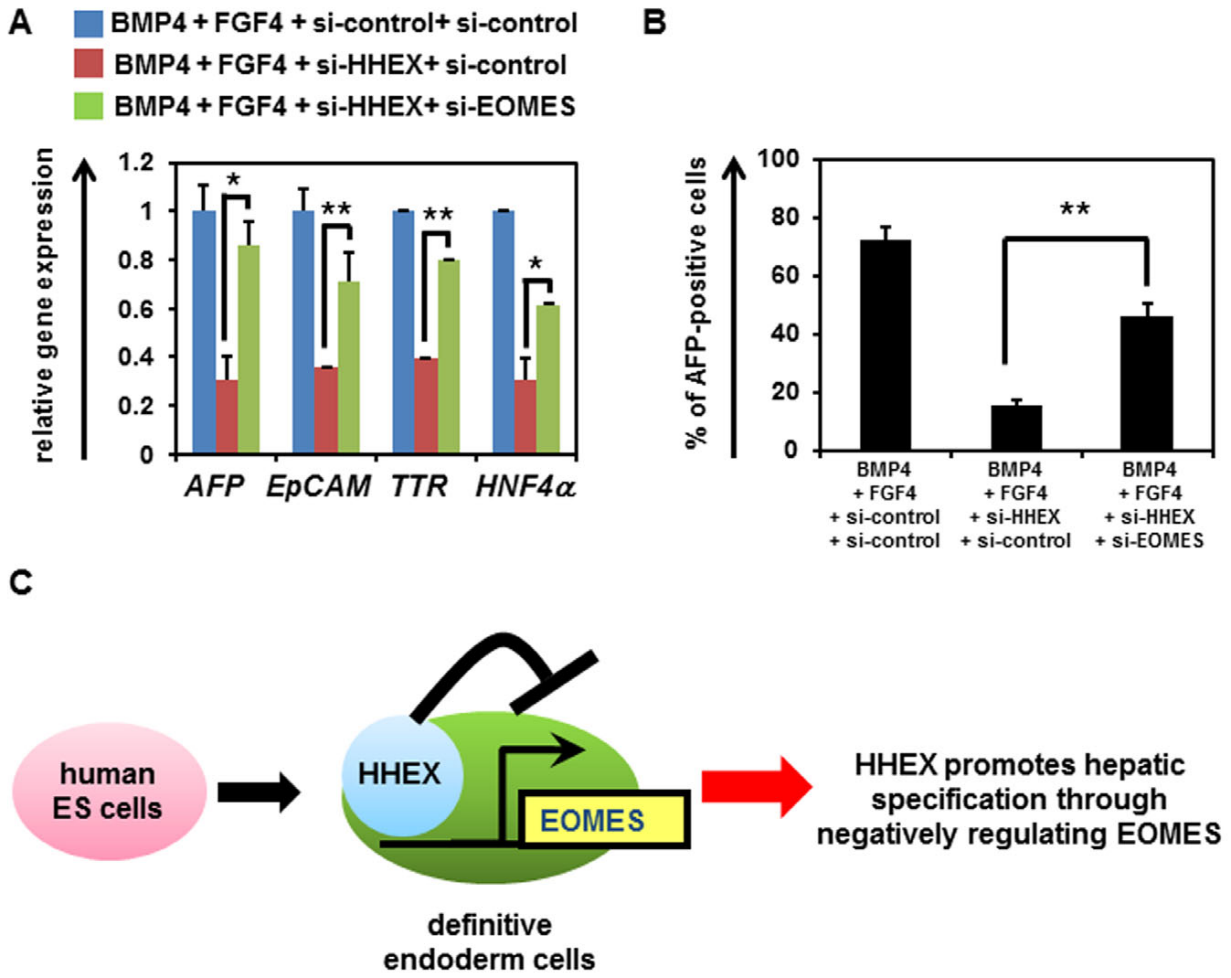
Hepatoblast differentiation is inhibited by EOMES, which functions downstream of HHEX. (**A**) The hESCs (H9) were differentiated into the DE cells according to the protocol described in the *Materials and Methods* section. The hESC-derived DE cells were transfected with 50 nM si-control, si-EOMES, or si-HHEX on day 4, and then cultured with the medium containing BMP4 and FGF4. The gene expression levels of hepatoblast markers (*AFP*, *EpCAM*, *TTR*, and *HNF4α*) were measured by real-time RT-PCR on day 9. The gene expression levels in si-control- and si-HHEX-transfected cells were taken as 1.0. (**B**) The percentage of AFP-positive cells was examined by FACS analysis on day 9. All data are represented as means ± SD (*n = *3). *p<0.05, **p<0.01. (**C**) HHEX promotes the hepatic specification from the hESC-derived DE cells by negatively regulating EOMES expression. A model of the hepatic specification from the hESC-derived DE cells by HHEX is presented. In the hESC-derived DE cells, HHEX represses EOMES expression. In this way, HHEX promotes the hepatic specification from the hESC-derived DE cells.

To examine whether EOMES functions downstream of HHEX in the hepatic specification from the DE cells, both HHEX and EOMES were knocked down in the DE cells, and then the gene expression profiles of hepatoblast markers were analyzed. The gene expression levels of hepatoblast markers were upregulated in both si-HHEX- and si-EOMES-transfected cells as compared with those in si-HHEX-transfected cells (**Fig. 5A**). Furthermore, the percentage of AFP-positive cells was also increased by double-knockdown of HHEX and EOMES (**Fig. 5B**). These results suggest that EOMES knockdown could promote the hepatic specification from the DE cells by HHEX knockdown. In conclusion, EOMES exerts downstream of HHEX in the hepatic specification from the DE cells.

## Discussion

The purpose of this study was to identify and characterize the target genes of HHEX in hepatic specification from DE to elucidate the functions of HHEX in this process. We clearly demonstrated that the expression of EOMES is directly suppressed by HHEX, and that EOMES is one of the crucial target genes of HHEX in the hepatic specification from the hESC-derived DE cells. We also showed that EOMES knockdown in the hESC-derived DE cells could rescue the si-HHEX-mediated inhibition of hepatic specification. Our findings indicate that promotion of the hepatic specification by HHEX in the hESC-derived DE cells would be mainly mediated by the repression of EOMES expression (**Fig. 5C**).

To explore direct target genes of HHEX in the hepatic specification, EOMES knockdown experiments were conducted (**Fig. 1**). The luciferase reporter assays (**Fig. 2B**) and ChIP-qPCR (**Fig. 3C**) indicated that HHEX represses EOMES expression by binding to the first intron of EOMES containing a putative HRE. It might be expected that HHEX recruits co-repressor proteins to repress EOMES expression because HHEX could negatively regulate the expressions of target genes such as *vascular endothelial growth factor (Vegf)* and *vascular endothelial growth factor receptor-1 (Vegfr-1)* by forming the co-repressor protein complexes [23]–[25]. Previous studies demonstrated that HHEX has three main domains, a repression domain, a DNA-binding domain, and an activation domain [26], and thus exerts both positive and negative effects on the target gene expressions. Taken together, these findings suggested that HHEX would repress EOMES expression through the function of its repression domain.

The results in **figure 4A** demonstrate that EOMES knockdown promoted hepatic specification in the presence of BMP4, but not FGF4. Because it was previously reported that FGF4 could induce the expression level of HHEX in the DE cells [27], FGF4 treatment in the DE cells would lead to downregulation of EOMES expression via the regulation of HHEX expression. Therefore, HHEX and EOMES might exert in the downstream of FGF4 in the hepatic specification. In addition, both BMP4 and FGF4 are necessary for hepatic specification (**Fig. 4A**). However, the functions of BMP4 in hepatic specification and the synergistic effect of BMP and FGF have not been sufficiently elucidated, and will need to be resolved in future studies.

Simultaneous knockdown of HHEX and EOMES in the hESC-derived DE cells led to rescue of the HHEX-mediated inhibition of the hepatic specification (**Fig. 5**). These results suggested that the majority of functions in the hepatic specification by HHEX may be caused by the repression of EOMES expression. EOMES is known to regulate numerous target genes related to DE differentiation, and thus the repression of EOMES expression might also promote other DE-derived lineage specifications, such as pancreatic specification. HHEX is known to regulate not only hepatic specification but also pancreatic specification [11]–[28]. Therefore, EOMES might also be a target gene of HHEX in pancreatic specification as well as in hepatic specification. Because the HHEX protein is known to interact with the HNF1α protein and synergistically upregulate the HNF1α target gene expression [15], it would be of interest to examine the relationship between HHEX and HNF1α in the hepatic specification from the hESC-derived DE cells. The proteomic analyses of HHEX protein in the hepatic specification from the hESC-derived DE cells might help to elucidate the functions of HHEX in this process.

## Conclusions

In summary, we showed that the homeobox gene HHEX promotes the hepatic-lineage specification from the hESC-derived DE cells through the repression of EOMES expression. Previously, we reported that transduction of SOX17, HNF4α, FOXA2 or HNF1α into the hESC-derived cells could promote efficient hepatic differentiation [16]–[18]. The direct target genes of these genes might be identified by using the strategy described here. Furthermore, identification of the genes targeted by functional genes in the various lineage differentiation models from hESCs will promote understanding of the intricate transcriptional networks that regulate human development.

## Supporting Information

File S1 Contains the following files: **Figure S1. Knockdown of HHEX in the DE cells by si-HHEX transfection.** (**A, B**) The hESCs (H9) were differentiated into the DE cells (day 4) according to the protocol described in *Materials and Methods* section. The DE cells were transfected with 50 nM si-control or si-HHEX on day 4. On day 6, the HHEX expression levels in si-control- or si-HHEX-transfected cells were examined by real-time RT-PCR (**A**) or Western blotting (**B**). The gene expression levels of *HHEX* in the si-control-transfected cells were taken as 1.0. All data are represented as means ± SD (*n = *3). ** p<0.01. **Figure S2. The percentage of AFP-positive cells or** ***EOMES*** **expression level was decreased or increased, respectively, by HHEX knockdown.** (**A, B**) The hESCs (H9) were differentiated into the DE cells according to the protocol described in the *Materials and Methods* section. The DE cells were transfected with 50 nM si-control or si-HHEX on day 4, 5, 6, or 7, and cultured in medium containing 20 ng/ml BMP4 and 20 ng/ml FGF4 until day 9. On day 9, the percentage of AFP-positive cells was measured by using FACS analysis to examine the hepatoblast differentiation efficiency (**A**). Also on day 9, the gene expression levels of EOMES in si-control- or si-HHEX-transfected cells were examined by real-time RT-PCR (**B**). The gene expression levels in the si-control-transfected cells were taken as 1.0. All data are represented as means ± SD (*n = *3). **p<0.01. **Figure S3. Both 1,000 bp and 4,000 bp 5′ UTR of EOMES have promoter activities.** Luciferase reporter assays were performed to examine whether 1,000 bp and 4,000 bp 5′ UTR of EOMES have promoter activity. HeLa cells were cotransfected with both 500 ng/well of firefly luciferase reporter plasmids (pControl-Luc, p5’ EOM-Luc, or pLong-5′ EOM-Luc), and 500 ng/well of internal control plasmids (pCMV-Renilla luciferase), and cultured for 72 hours. The luciferase activities in the cells were measured by using Dual Luciferase Assay System (Promega) according to the manufacturer’s instructions. Firefly luciferase activities in the cells were normalized by the measurement of renilla luciferase activities. The RLU in the pControl-Luc-transfected cells was assigned a value of 1.0. All data are represented as means ± SD (*n = *3). *, p<0.05. **Figure S4. Knockdown of EOMES in the DE cells by si-EOMES transfection.** (**A, B**) The hESCs (H9) were differentiated into the DE cells (day 4) according to the protocol described in *Materials and Methods* section. The DE cells were transfected with 50 nM si-control or si-EOMES on day 4. On day 6, the EOMES expression levels in si-control- or si-EOMES-transfected cells were examined by real-time RT-PCR (**A**) or Western blotting (**B**). The gene expression levels of *EOMES* in the si-control-transfected cells were taken as 1.0. All data are represented as means ± SD (*n = *3). ** p<0.01. **Figure S5.** **Hepatoblast differentiation was promoted by knockdown of EOMES.** The hESCs (H9) were differentiated into the DE cells according to the protocol described in the *Materials and Methods* section. The hESC-derived DE cells were transfected with 50 nM si-control or si-EOMES on day 4, 5, 6, or 7, and then cultured in medium containing BMP4 or FGF4. The percentage of AFP-positive cells was examined by FACS analysis on day 9. All data are represented as means ± SD (*n = *3). **p<0.01. **Figure S6.** **The** ***EOMES*** **or** ***HHEX*** **expression level was suppressed or increased, respectively, in the presence of FGF4.** The hESCs (H9) were differentiated into the DE cells according to the protocol described in the *Materials and Methods* section. The hESC-derived DE cells were cultured in medium containing BMP4 or FGF4 until day 9. The gene expression levels of *EOMES*, *HHEX*, or *AFP* in the non-treated cells (control) were taken as 1.0. All data are represented as means ± SD (*n = *3). **p<0.01 (compared with control).
(PDF)

File S2 Contains the following files: **Table S1.** List of primers used in this study. **Table S2.** List of antibodies used in this study.
(DOC)
