# Supplementary figures and images for: HHEX Promotes Hepatic-Lineage Specification through the Negative Regulation of Eomesodermin
Source: PLoS One. 2014 Mar 20;9(3):e90791. doi: 10.1371/journal.pone.0090791 (PMC3961246; doi:10.1371/journal.pone.0090791)

Figure S1

A

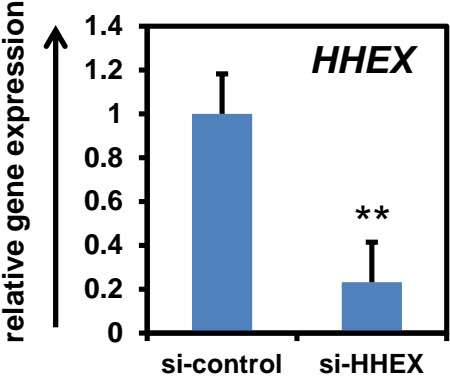

B

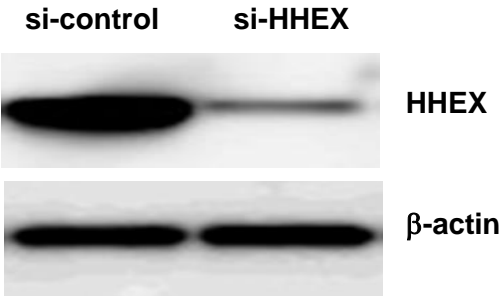

Figure S2

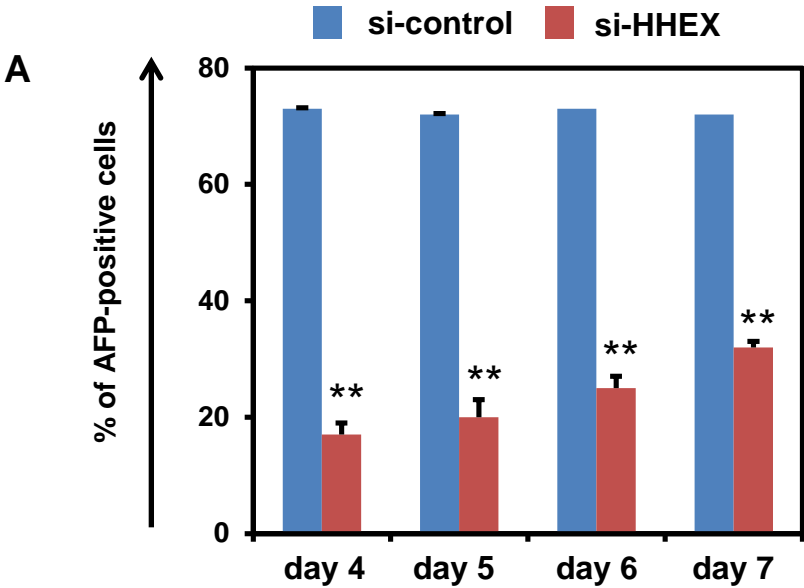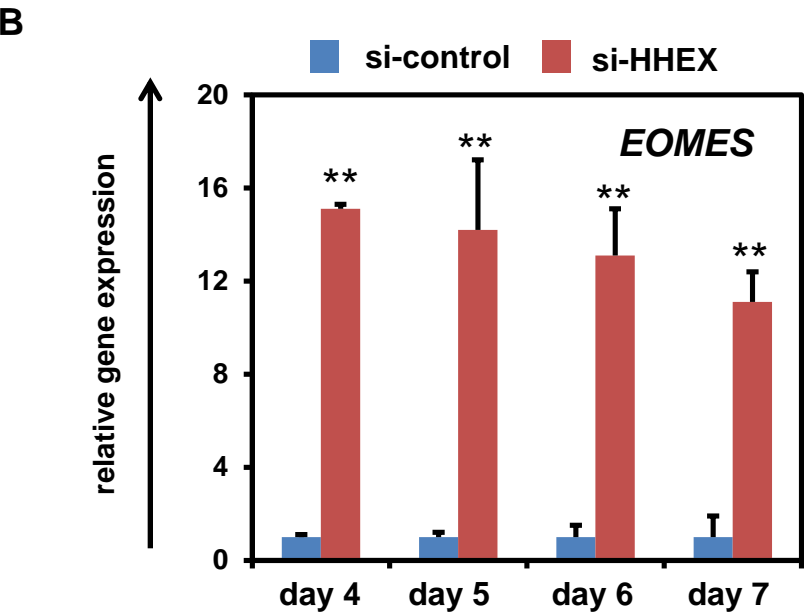

Figure S3

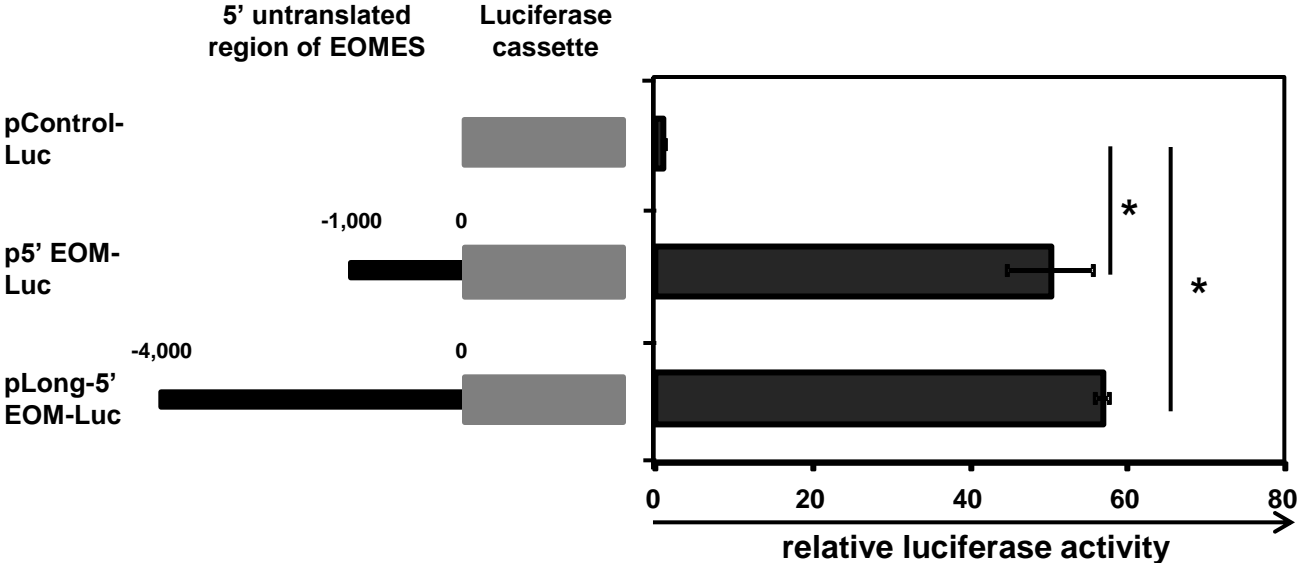

Figure S4

A

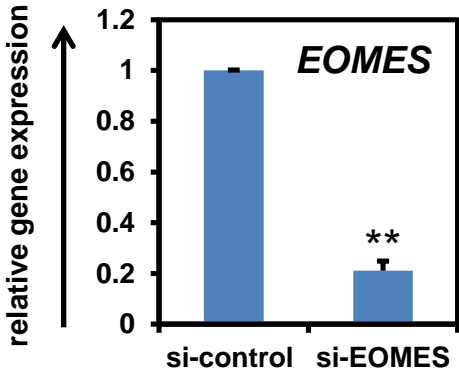

B

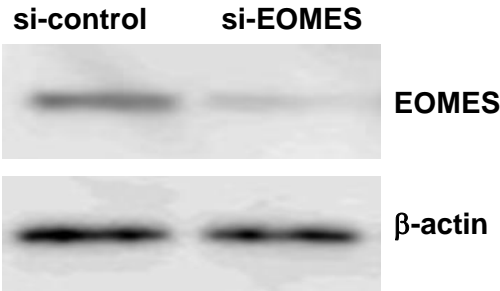

Figure S5

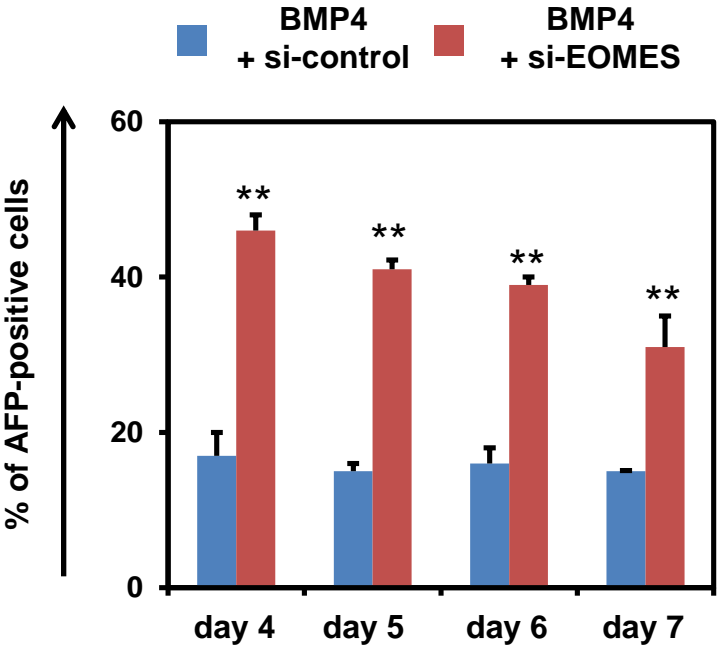

Figure S6

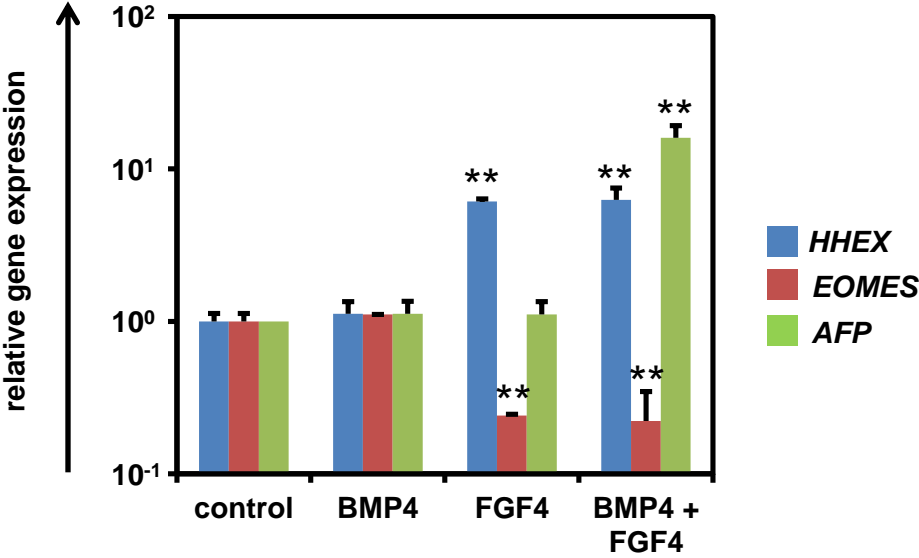

Supplement: File S1 — Contains the following files: Figure S1. Knockdown of HHEX in the DE cells by si-HHEX transfection. (A, B) The hESCs (H9) were differentiated into the DE cells (day 4) according to the protocol described in Materials and Methods section. The DE cells were transfected with 50 nM si-control or si-HHEX on day 4. On day 6, the HHEX expression levels in si-control- or si-HHEX-transfected cells were examined by real-time RT-PCR (A) or Western blotting (B). The gene expression levels of HHEX in the si-control-transfected cells were taken as 1.0. All data are represented as means ± SD (n = 3). ** p<0.01. Figure S2. The percentage of AFP-positive cells or EOMES expression level was decreased or increased, respectively, by HHEX knockdown. (A, B) The hESCs (H9) were differentiated into the DE cells according to the protocol described in the Materials and Methods section. The DE cells were transfected with 50 nM si-control or si-HHEX on day 4, 5, 6, or 7, and cultured in medium containing 20 ng/ml BMP4 and 20 ng/ml FGF4 until day 9. On day 9, the percentage of AFP-positive cells was measured by using FACS analysis to examine the hepatoblast differentiation efficiency (A). Also on day 9, the gene expression levels of EOMES in si-control- or si-HHEX-transfected cells were examined by real-time RT-PCR (B). The gene expression levels in the si-control-transfected cells were taken as 1.0. All data are represented as means ± SD (n = 3). **p<0.01. Figure S3. Both 1,000 bp and 4,000 bp 5′ UTR of EOMES have promoter activities. Luciferase reporter assays were performed to examine whether 1,000 bp and 4,000 bp 5′ UTR of EOMES have promoter activity. HeLa cells were cotransfected with both 500 ng/well of firefly luciferase reporter plasmids (pControl-Luc, p5’ EOM-Luc, or pLong-5′ EOM-Luc), and 500 ng/well of internal control plasmids (pCMV-Renilla luciferase), and cultured for 72 hours. The luciferase activities in the cells were measured by using Dual Luciferase Assay System (Promega) ac [file pone.0090791.s001.pdf]
