# Supplementary material for: HHEX Promotes Hepatic-Lineage Specification through the Negative Regulation of Eomesodermin
Source: PLoS One. 2014 Mar 20;9(3):e90791. doi: 10.1371/journal.pone.0090791 (PMC3961246; doi:10.1371/journal.pone.0090791)
Supplement: File S2 — Contains the following files: Table S1. List of primers used in this study. Table S2. List of antibodies used in this study. (DOC) [file pone.0090791.s002.doc]

**Table S1.**  **List of primers used in this study**

Real-time RT-PCR

| Gene Symbol | Primers (forward/reverse; 5' to 3') |
| --- | --- |
| AFP | TGGGACCCGAACTTTCCA/GGCCACATCCAGGACTAGTTTC |
| CDX2 | TCCGTGTACACCACTCGATATT/GGAACCTGTGCGAGTGGAT |
| EOMES | CAGCACCACCTCTACGAACA/CGCCACCAAACTGAGATGAT |
| EpCAM | AATCGTCAATGCCAGTGTACTT/TCTCATCGCAGTCAGGATCATAA |
| FOXA2 | GCGACCCCAAGACCTACAG/GGTTCTGCCGGTAGAAGGG |
| GAPDH | GGTGGTCTCCTCTGACTTCAACA/GTGGTCGTTGAGGGCAATG |
| GATA4 | CATCAAGACGGAGCCTGGCC/TGACTGTCGGCCAAGACCAG |
| GATA6 | CCATGACTCCAACTTCCACC/ACGGAGGACGTGACTTCGGC |
| GSC | TCTCAACCAGCTGCACTGTC/CGTTCTCCGACTCCTCTGAT |
| HHEX | CACCCGACGCCCTTTTACAT/GAAGGCTGGATGGATCGGC |
| HNF4α | CGTCATCGTTGCCAACACAAT/GGGCCACTCACACATCTGTC |
| KLF5 | TCCCAGGTACACTTGTATGGC/ACCCTGGTTGCACAAAAGTT |
| NANOG | AGAAGGCCTCAGCACCTAC/GGCCTGATTGTTCCAGGATT |
| NKX2-2 | CCGGGCCGAGAAAGGTATG/GTTTGCCGTCCCTGACCAA |
| NKX6-1 | GGACTGCCACGCTTTAGCA/TGGGTCTCGTGTGTTTTCTCT |
| OCT3/4 | CTTGAATCCCGAATGGAAAGGG/GTGTATATCCCAGGGTGATCCTC |
| PDX1 | CGTCCGCTTGTTCTCCTC /CCTTTCCCATGGATGAAGTC |
| PROX1 | TTGACATTGGAGTGAAAAGGACG/TGCTCAGAACCTTGGGGATTC |
| SOX17 | GTGGACCGCACGGAATTTG/GAGGCCCATCTCAGGCTTG |
| TTR | TCATCGTCTGCTCCTCCTCT/AGGTGTCATCAGCAGCCTTT |

**ChIP-qPCR**

| Gene Symbol | Primers (forward/reverse; 5' to 3') |
| --- | --- |
| EOMES | GGTACTTTTCAAAATGGTGCA/GCCTGTGCAAGGGAATAGAATC |
| β-ACTIN | CAGGGCTTCTTGTCCTTTCC/GTAGAAGGTGTGGTGCCAGA |

**Table S2. List of antibodies used in this study**

| Gene Symbol | Type | Company |
| --- | --- | --- |
| AFP | mouse | Cell Signaling |
| Alexa Fluor 488 anti-mouse IgG | mouse | Invitrogen |
| β-actin | mouse | Sigma |
| EOMES | mouse | eBioscience |
| HHEX | rabbit | Abcam |
| horseradish peroxidaseconjugated anti-rabbit IgG |  | Cell Signaling |
| horseradish peroxidaseconjugated anti-mouse IgG |  | Cell Signaling |
| mIgG | goat | Santa Cruz Biotechnology |
